# The INSPIRE-T longitudinal observational study centered on intrinsic capacity: baseline data

**DOI:** 10.1093/gerona/glaf181

**Published:** 2025-08-19

**Authors:** Sophie Guyonnet, Claudie Hooper, Christelle Cantet, Nicola Coley, Sandrine Andrieu, Bruno Vellas

**Affiliations:** Department of Geriatrics, Institute Hospital-Universitaire (IHU) HealthAge, Gérontopôle, CHU Toulouse, Toulouse, France; CERPOP, INSERM UMR, University of Toulouse, Toulouse, France; Department of Geriatrics, Institute Hospital-Universitaire (IHU) HealthAge, Gérontopôle, CHU Toulouse, Toulouse, France; Department of Geriatrics, Institute Hospital-Universitaire (IHU) HealthAge, Gérontopôle, CHU Toulouse, Toulouse, France; CERPOP, INSERM UMR, University of Toulouse, Toulouse, France; CERPOP, INSERM UMR, University of Toulouse, Toulouse, France; Department of Epidemiology and Public Health, Toulouse University Hospital, Toulouse, France; CERPOP, INSERM UMR, University of Toulouse, Toulouse, France; Department of Epidemiology and Public Health, Toulouse University Hospital, Toulouse, France; Department of Geriatrics, Institute Hospital-Universitaire (IHU) HealthAge, Gérontopôle, CHU Toulouse, Toulouse, France; CERPOP, INSERM UMR, University of Toulouse, Toulouse, France; Medical Sciences Section

**Keywords:** WHO, ICOPE, Aging, Biomarkers

## Abstract

**Background:**

The “INStitute for Prevention,” “healthy agIng,” and “medicine REjuvenative” “Translational” (INSPIRE-T) study is a 10-year observational study. The primary objective of which is to study trajectories of aging across lifespan from the perspective of intrinsic capacity (IC) through deep clinical and biological phenotyping.

**Methods:**

IC was assessed in the INSPIRE-T cohort stratified by age (10-year age groups) using the World Health Organization (WHO) integrated care for older people (ICOPE) program. Biospecimens (biofluids, dental plaque, nasopharyngeal swabs, stools, hair follicles, and skin biopsies) were also collected for biomarker studies and drug-target identification for the prediction and prevention of IC declines respectively.

**Results:**

The INSPIRE-T baseline data is presented including demographic, health, anthropometric, lifestyle, and IC characteristics of the cohort. The open cohort (ongoing recruitment) currently consists of 1109 participants with data available at baseline (61.9% female) aged from 20 to 102 years old (mean age ± standard deviation: 62.4 ± 19.0 years). The most prevalent IC abnormalities identified using ICOPE *Step 1*. screening appeared to be in vision (89.3%), audition (30.5%), and cognition (24.7%) in the total baseline population, while locomotor capacity abnormalities (5.4%) appeared to be the least frequent. Sex-specific differences were observed for the domains of psychological well-being, audition, vitality in 30-39-year olds and vision in subjects ≤39 years old.

**Conclusions:**

The ultimate goal of INSPIRE-T is to enable the identification of individuals at risk of IC declines in order to implement personalized preventive interventions to promote healthy aging.

## Introduction

In the first World Report on Ageing and Health, the World Health Organization (WHO) redefined “*healthy aging*” centered on the concept of intrinsic capacity (IC).^1^^,^^2^ IC is the composite of an individual’s physical and mental capacities that in concert with environmental factors govern functional ability.^2^ IC comprises six interconnected domains: *cognition, locomotor capacity, psychological well-being, audition, vision* and *vitality.* IC can be assessed using the recommendations in the WHO Integrated Care for Older People (ICOPE) handbook (five-step process in the original version).^3^ Research on IC is still in its infancy, only limited studies have investigated the associations of IC with health outcomes and functional ability over extended periods of time using the ICOPE tool.^4-6^ Thus, the “INStitute for Prevention,” “healthy agIng,” and “medicine REjuvenative,” “Translational” (INSPIRE-T) longitudinal observational study was specifically designed to address this research gap through deep and frequent clinical and biological phenotyping.

INSPIRE-T comprises community-dwelling adults with varying degrees of functional capacity and has a pre-planned follow-up of 10 years. The assessment of IC was implemented at study baseline (and at subsequent follow-ups) using the ICOPE program (*Steps 1 and 2*)^3^ in combination with numerous other clinical examinations and biospecimen collection in order to comprehensively capture the biology of aging. The INSPIRE-T biospecimens will enable the future identification of biomarkers of IC status and putative drug targets that protect against IC declines. Here, we describe the baseline data from the INSPIRE-T study with its focus on IC and lifestyle factors as potentially modifiable variables to mitigate adverse IC trajectories. These data define the start of a novel study that will enrich our understanding of the aging process and propel Geroscience research to new heights.

## Methods

### Study description

INSPIRE-T is a single-centered observational study with a 10-year follow-up. The rationale, methodology, and detailed objectives of INSPIRE-T are reviewed elsewhere.^7^^,^^8^ The study was approved by the French Ethics Committee in Rennes (CPP Ouest V) and the study was registered at http://clinicaltrials.gov (NCT04224038). Recruitment to INSPIRE-T started in October 2019, and all participants provided signed and informed consent with the vast majority of subjects (*n* = 1000) being recruited by December 2021. Participants of INSPIRE-T are stratified into 10-year age groups (with the oldest age group being ≥80 years old) with oversampling of subjects aged ≥70 years old. Recruitment remains open to maintain a minimum pool of 1 000 participants in the study.

### Target population

The baseline population consists of 1 109 subjects with available data. Participants were recruited from the city of Toulouse and included men and women aged ≥20 years old without an imposed upper age limit. There were also no strict inclusion or exclusion criteria in INSPIRE-T. Only people deprived of their liberty through judicial decision were excluded or those with a life expectancy of ≤5 years. A life expectancy of ≤2 years was classed as an exclusion criterion in the case of frail older adults or subjects aged >80 years.

### Data collection

Data was collected at study baseline and during follow-up visits (annual visits and additional visits in the event of IC declines).^7^ Additionally, participants had IC (ICOPE *Step 1*, original version) monitored at 4-monthly intervals throughout the first year of the study and thereafter every 6 months either through the use of an application (ICOPE Monitor), an online platform or through a telephone call with a research nurse.^3^^,^^9^ Baseline data included the following items and clinical examinations:

- Demographic information such as marital status, education, and living status.
- General examination including medical history, vaccination record, medication taken, health conditions, body mass index (BMI), waist and hip circumference, heart rate, and blood pressure (BP).
- Lifestyle information including: subjectively measured physical activity and sedentary time assessed using the International Physical Activity Questionnaire (IPAQ),^10^ smoking status, dietary information (data not shown) and alcohol consumption assessed using the Food frequency questionnaire (FFQ)^11^ and self-reported sleep duration. Fatigue and social isolation were evaluated using Patient-Reported Outcomes Measurement Information System (PROMIS)^12^ questionnaires (short form 8a for both).
- ICOPE *Step 1*. screening for data on IC domains.^3^  ***Locomotor capacity*** was measured by time (in seconds) required to perform five-chair rises. An abnormality was recorded when the time required to complete the test is ≥ 14 seconds for subjects <80 years old or ≥16 seconds for subjects ≥80 years old or number of chair rises <5. ***Psychological well-being*** was measured by the following questions: Over the past 2 weeks, have you been bothered by: 1. Feeling down, depressed or hopeless? 2. Having little interest or pleasure in doing things? A «yes» response for at least one question determined an abnormality. ***Cognition*** was measured by the three-word remember test and the following four orientation questions: Which year is it? What month is it? What day of the month is it? What day of the week is it? from the Mini Mental State Examination (MMSE).^13^ An abnormality was recorded if an individual was unable to remember at least one word or if they provided an incorrect response to an orientation question. ***Vitality*** was measured by the following questions: Have you unintentionally lost more than 3 kg over the last 3 months? Have you experienced loss of appetite? A «yes» response for at least one question determined an abnormality. ***Vision*** was measured by the following questions: Do you have any problems with your eyes? Difficulties in seeing far, reading, eye diseases or are you currently under medical treatment? A «yes» response determined an abnormality. Audition was measured using the whisper test. The evaluator whispers a two-syllable word and asks the person to repeat the word. The evaluator then tests the other ear using a different word. Not repeating the correct words determined an abnormality.
- ICOPE *Step 2*. assessments for the deeper characterization of IC domains^3^ with abnormalities defined according to the predefined cut-offs subsequently described. ***Cognition*** was assessed using the MMSE (score: 0-30)^13^ with a cut-off of <26 used as an indicator of cognitive impairment in highly-educated people.^14^ To provide a measure of ***vitality***, nutritional status was evaluated with the Mini Nutritional Assessment (MNA: score 0-30) with a cut-off of ≤23.5 indicating a risk of malnutrition.^15^ To evaluate ***locomotor capacity***, physical performance was measured with the Short Physical Performance Battery (SPPB: score 0-12)^16^ with a cut-off of <10 suggestive of mobility limitations.^17^ To provide a measure of ***psychological well-being***, depressive symptoms were assessed using the Patient Health Questionnaire (PHQ-9: score 0-27) with a cut-off of >4 indicative of depression^18^ and ***vision*** was evaluated using the WHO simple eye chart. Audiometry for the deeper assessment of hearing was not performed at baseline (audiometry was performed annually from *year 1*).

Other tests not stipulated in the ICOPE program, but potentially applicable for the exploratory assessment of IC and its underlying biology included:

- The Cognitive Function Instrument (CFI)^19^ for the subjective assessment of cognition (Table S1).
- Functional performance measured using Activities of Daily Living (ADL)^20^ and Instrumental Activities of Daily Living (IADL) scales^21^ (Table S1) to provide measures of locomotor capacity and cognition for more complex IADL.
- PROMIS mobility questionnaire (custom 14 items questionnaire from the PROMIS mobility v2.1 items bank) and the maximum number of chair rises in 30 seconds^22^ (Table S1) enabling the further characterization of locomotor capacity.
- The Amsler grid (Table S1) to explore vision (macular health).
- Oral status assessed using the Oral Health Assessment Tool (OHAT)^23^ (Table S1) given its potential impact on nutritional status and in turn vitality.
- Fried’s frailty phenotype^24^ (Table S2) due to its inverse association with vitality in older adults.
- Dual-energy X-ray absorptiometry (DXA) measurements for body composition and sarcopenia were performed in a subset of participants (*n* = 850) (with follow-up at *year 2* then every 5 years) (Table S3) due to their association with vitality and locomotor capacity.
- Whole body and brain MRI in a subset of participants (*n* = 120: data not shown) to provide further information on vitality and cognition respectively.
- Maximum oxygen consumption (VO_2max_) as a measure of cardiovascular fitness as well as isokinetic muscle strength (Cybex) were assessed between baseline and *year 2* in a subset of participants (Table S4, *n* = 279 and *n* = 286 respectively) to further explore vitality.
- For participants <70 years old, cognition was also assessed using: the free and total recall of the Free and Cued Selective Reminding Test (FCSRT),^25^ the 10 MMSE orientation items,^26^ the Digit Symbol Substitution Test (DSST) from the Wechsler Adult Intelligence Scale-Revised^27^ and the Category Naming Test (CNT)^28^ (2-minute category fluency in animals) (Table S5). Cognition was also assessed in this subset using the MAPT Preclinical Alzheimer Cognitive Composite (MAPT-PACC), which is the mean of the z-scores of the four aforementioned cognitive tests^29^ (Table S5). The MAPT-PACC score provides a sensitive measure of early cognitive loss.^29^

### Biobank

Biospecimens including blood, urine, saliva, and dental plaque were collected at baseline then annually (Table S6). Nasopharyngeal swabs, stools, hair follicles, skin surface samples, and skin biopsies were collected optionally at baseline. Stools and hair follicle samples will be subsequently collected every 2 years.

### Data sharing

Data will be shared with the scientific community following curation no more than 1 year after study-team exclusive access.

### Statistics

Descriptive statistics are reported as means ± standard deviation (SD) or as absolute values and percentages. Kruskal–Wallis or chi square tests for continuous or categorical variables respectively were used to assess differences in ICOPE measurements between age groups and *P*-values of < .05 were considered statistically significant. Statistics were performed using SAS software version 9.4 (SAS Institute Inc, Cary, NC).

## Results

### Baseline demographic description

Demographic characteristics of the study participants stratified into 10-year age groups are shown in Table 1. The mean age of the 1 109 participants at baseline was 62.4 ± 19.0 years. Participants’ age ranged from 20 to 102 years with 42.9% of the participants aged over 70 years. The cohort was 61.9% female, and participants had a high educational level with 71.9% declaring university attendance. Approximately 30% of the participants lived alone and were single, divorced or widowed.

**Table 1.** Baseline demographic characteristics (*n* = 1109).

Characteristic	Total	20-29 years	30-39 years	40-49 years	50-59 years	60-69 years	70-79 years	≥80 years
	(*n* = 1109)	(*n* = 83)	(*n* = 91)	(*n* = 108)	(*n* = 145)	(*n* = 206)	(*n* = 225)	(*n* = 251)
**Age (years), mean (SD)**	62.4 (19.0)	24.4 (2.8)	34.3 (3.0)	45.6 (2.7)	54.5 (2.8)	64.3 (2.6)	74.4 (3.0)	84.6 (4.0)
**Sex (female), *n* (%)**	687 (61.9%)	57 (68.7%)	57 (62.6%)	73 (67.6%)	102 (70.3%)	129 (62.6%)	126 (56.0%)	143 (57.0%)
**Educational level, *n* (%)**								
**No diploma or primary certificate**	50 (4.5%)	0 (0.0%)	0 (0.0%)	0 (0.0%)	0 (0.0%)	4 (1.9%)	11 (4.9%)	35 (14.3%)
**Secondary education**	101 (9.2%)	1 (1.2%)	0 (0.0%)	2 (1.9%)	6 (4.1%)	22 (10.7%)	23 (10.3%)	47 (19.3%)
**High school diploma**	158 (14.4%)	1 (1.2%)	2 (2.2%)	12 (11.1%)	22 (15.2%)	35 (17.0%)	49 (21.9%)	37 (15.2%)
**University level**	792 (71.9%)	81 (97.6%)	89 (97.8%)	94 (87.0%)	117 (80.7%)	145 (70.4%)	141 (62.9%)	125 (51.2%)
***Data missing for, (n)***	*8*	*0*	*0*	*0*	*0*	*0*	*1*	*7*
**Living Status, *n* (%)**								
**Living alone**	313 (28.8%)	29 (36.7%)	16 (18.4%)	17 (16.0%)	34 (23.6%)	55 (26.8%)	62 (27.9%)	100 (41.3%)
**Living with spouse, family or other**	749 (69.1%)	50 (63.3%)	71 (81.6%)	89 (84.0%)	110 (76.4%)	150 (73.2%)	155 (69.8%)	124 (51.3%)
**Living in a residence for older adults**	23 (2.1%)	0 (0.0%)	0 (0.0%)	0 (0.0%)	0 (0.0%)	0 (0.0%)	5 (2.3%)	18 (7.4%)
***Data missing for, (n)***	*24*	*4*	*4*	*2*	*1*	*1*	*3*	*9*
**Marital Status, *n* (%)**								
**Married (or in a couple)**	710 (64.4%)	51 (61.4%)	69 (75.8%)	76 (70.4%)	95 (65.5%)	147 (71.4%)	157 (69.8%)	115 (47.1%)
**Single**	134 (12.2%)	32 (38.6%)	20 (22.0%)	20 (18.5%)	15 (10.3%)	18 (8.7%)	8 (3.6%)	21 (8.6%)
**Divorced**	141 (12.8%)	0 (0.0%)	2 (2.2%)	11 (10.2%)	33 (22.8%)	34 (16.5%)	37 (16.4%)	24 (9.8%)
**Widowed**	117 (10.6%)	0 (0.0%)	0 (0.0%)	1 (0.9%)	2 (1.4%)	7 (3.4%)	23 (10.2%)	84 (34.4%)
***Data missing for, (n)***	*7*	*0*	*0*	*0*	*0*	*0*	*0*	*7*

Data are expressed as mean ± standard deviation (SD) or as absolute values/percentages. Missing data from participants (*n*) specified in categories lacking data.

### Baseline health conditions, medication, and anthropometric measures

Health conditions, medication, and anthropometric measures are shown in Table 2. The most prominent health conditions were hypertension (27.6%), hypercholesterolemia (19.5%), depression (17.1%), and arthritis (11.7%). Participants took on average about three items of prescription medication per day (mean 2.6 ± 3.1) with those participants aged over 80 years taking around five items of prescription medication on average per day (mean 5.3 ± 3.4). The mean BMI of the total population was 25.2 ± 4.3 kg/m^2^. The mean waist and hip circumference of the total population was 88.3 ± 13.1 cm (range: from 77.8 ± 10.0 to 94.2 ± 12.0 cm) and 99.7 ± 8.9 cm (range: from 96.9 ± 7.5 to 100.5 ± 8.6 cm) respectively. The mean heart rate of the total population was 67.9 ± 11.6 beats/min with the highest heart rate observed in the youngest age group (71.8 ± 13.0 beats/min). The mean systolic BP of the total population was 131.1 ± 20.7 mm Hg (range: 115.8 ± 12.0 mm Hg to 145.2 ± 21.5 mm Hg) with the highest systolic BP being observed in the oldest age-group. The mean diastolic BP was 73.0 ± 10.8 mm Hg in the total population (range: from 67.6 ± 9.6 to 75.2 ± 11.0 mm Hg).

**Table 2.** Baseline health conditions, medication, and anthropometric measures (*n* = 1109).

Characteristic	Total	20-29 years	30-39 years	40-49 years	50-59 years	60-69 years	70-79 years	≥80 years
	(*n* = 1109)	(*n* = 83)	(*n* = 91)	(*n* = 108)	(*n* = 145)	(*n* = 206)	(*n* = 225)	(*n* = 251)
**Health conditions, *n* (%)**								
**Hypertension**	306 (27.6%)	1 (1.2%)	1 (1.1%)	4 (3.7%)	11 (7.6%)	36 (17.5%)	94 (41.8%)	159 (63.3%)
**Hypercholesterolemia**	216 (19.5%)	1 (1.2%)	2 (2.2%)	3 (2.8%)	12 (8.3%)	40 (19.4%)	74 (32.9%)	84 (33.6%)
***Data missing for, (n)***	*2*	*0*	*0*	*0*	*1*	*0*	*0*	*1*
**Depression**	190 (17.1%)	5 (6.0%)	11 (12.1%)	15 (13.9%)	24 (16.6%)	30 (14.6%)	47 (20.9%)	58 (23.2%)
***Data missing for, (n)***	*1*	*0*	*0*	*0*	*0*	*0*	*0*	*1*
**Arthritis**	130 (11.7%)	0 (0.0%)	0 (0.0%)	1 (0.9%)	4 (2.8%)	16 (7.8%)	40 (17.8%)	69 (27.7%)
***Data missing for, (n)***	*2*	*0*	*0*	*0*	*0*	*0*	*0*	*2*
**Medication (per day), mean (SD)**	2.6 (3.1)	0.6 (0.9)	0.6 (1.1)	1.1 (2.1)	1.2 (1.7)	1.8 (2.1)	3.5 (3.4)	5.3 (3.4)
**Body mass index (kg/m^2^), mean (SD)**	25.2 (4.3)	22.8 (3.6)	24.7 (4.9)	23.9 (3.6)	24.7 (3.7)	25.2 (4.2)	26.2 (4.6)	26.1 (4.2)
***Data missing for, (n)***	*3*	*0*	*0*	*0*	*0*	*0*	*1*	*2*
**Waist circumference (cm), mean (SD)**	88.3 (13.1)	77.8 (10.0)	83.0 (12.3)	82.1 (10.7)	86.1 (11.8)	87.7 (12.2)	92.6 (13.3)	94.2 (12.0)
***Data missing for, (n)***	*3*	*0*	*0*	*0*	*0*	*0*	*0*	*3*
**Hip circumference (cm), mean (SD)**	99.7 (8.9)	96.9 (7.5)	100.3 (10.2)	98.2 (7.3)	99.8 (8.3)	99.5 (8.8)	100.5 (8.6)	100.2 (10.1)
***Data missing for, (n)***	*3*	*0*	*0*	*0*	*0*	*0*	*0*	*3*
**Heart rate (beats/min), mean (SD)**	67.9 (11.6)	71.8 (13.0)	67.6 (12.1)	66.8 (10.1)	65.8 (8.7)	65.6 (10.3)	68.7 (13.3)	69.5 (11.7)
***Data missing for, (n)***	*4*	*0*	*0*	*0*	*0*	*1*	*0*	*3*
**Blood pressure recumbent, (mm Hg)**								
**Systolic, mean (SD)**	131.1 (20.7)	115.9 (10.9)	115.8 (12.0)	117.6 (13.1)	121.1 (15.9)	130.9 (16.4)	140.4 (19.2)	145.2 (21.5)
***Data missing for, (n)***	*3*	*0*	*0*	*0*	*0*	*1*	*0*	*2*
**Diastolic, mean (SD)**	73.0 (10.8)	68.8 (9.0)	67.6 (9.6)	71.0 (10.4)	72.6 (10.5)	73.9 (10.9)	75.2 (11.0)	74.9 (10.8)
***Data missing for, (n)***	*3*	*0*	*0*	*0*	*0*	*1*	*0*	*2*

Data are expressed as mean ± standard deviation (SD) or as absolute values/percentages. Missing data from participants (*n*) specified in categories lacking data.

### Baseline lifestyle characteristics

Baseline lifestyle characteristics of the population are presented in Table 3. Adherence to WHO physical activity recommendations (≥150 min/week moderate-intensity physical activity) was 82% in the total cohort. It appeared that the lowest percentage of people accomplishing this exercise goal was observed in the oldest age-group (68.4%). The mean number of hours spent seated representative of sedentary behavior was 6.8 ± 2.9 hours in the total population, and 43.9% of all participants spent ≥7 hours seated. It appeared that the time spent seated was greatest for those aged 20-59 years old. A small minority (6.7%) of the total population exceeded French health recommendations on alcohol consumption (>2 standard drinks per day). It appeared that the oldest age group followed by those subjects in the 40-49 years age group contained the greatest percentage of people exceeding these alcohol recommendations. Approximately 60% of the total population had never smoked. The youngest age bracket seemed to have the highest prevalence of subjects that had never smoked (81.9%). Current smokers represented around 8% of the total population with the highest prevalence of smokers appearing to be found in the 50-59 years age group. Approximately 60% of the population exhibited normal sleep duration of ≥7 hours and <9 hours.^30^ Short-sleep duration of <7 hours seemed most prevalent in the 50-59 years age group, while long-sleep duration ≥9 hours appeared more prevalent in the oldest age-group. The mean PROMIS fatigue T-scores across all age groups were below the reference population mean of 50 for the United States (mean T-score range: 42.1–47.4). The fatigue scores appeared to be the highest in the oldest age group with 24.9% of people in this age group deemed fatigued (T-score ≥ 55). Similarly, the mean PROMIS social isolation T-scores were below the reference population mean (50 for the United States) across all age groups (mean T-score range: 39.2–42.0). The highest prevalence of social isolation (T-score ≥ 55) was observed in the 30-39 years age group (8.1%).

**Table 3.** Baseline lifestyle characteristics (*n* = 1109).

Lifestyle characteristics	Total	20-29 years	30-39 years	40-49 years	50-59 years	60-69 years	70-79 years	≥80 years
	(*n* = 1109)	(*n* = 83)	(*n* = 91)	(*n* = 108)	(*n* = 145)	(*n* = 206)	(*n* = 225)	(*n* = 251)
**Subjectively measured physical activity^a^**								
**Min/day, mean (SD)**	110.9 (100.5)	130.0 (101.5)	113.0 (95.5)	124.0 (98.5)	120.7 (114.8)	142.4 (118.3)	107.6 (88.0)	69.0 (72.1)
**≥150 min/week, *n* (%)**	828 (82.0%)	71 (86.6%)	73 (83.9%)	90 (86.5%)	112 (83.6%)	157 (87.7%)	171 (85.9%)	154 (68.4%)
**<150 min/week, *n* (%)**	182 (18.0%)	11 (13.4%)	14 (16.1%)	14 (13.5%)	22 (16.4%)	22 (12.3%)	28 (14.1%)	71 (31.6%)
***Data missing for, (n)***	*99*	*1*	*4*	*4*	*11*	*27*	*26*	*26*
**Sedentary behavior**								
**Hours/day seated, mean (SD)**	6.8 (2.9)	8.4 (3.2)	7.7 (3.2)	7.5 (2.8)	7.6 (3.2)	5.9 (2.7)	5.8 (2.2)	6.7 (2.9)
**<7 hours/day, *n* (%)**	601 (56.1%)	25 (30.5%)	36 (39.6%)	44 (41.1%)	62 (42.8%)	148 (72.5%)	151 (69.9%)	135 (59.5%)
**≥7 hours/day, *n* (%)**	471 (43.9%)	57 (69.5%)	55 (60.4%)	63 (58.9%)	83 (57.2%)	56 (27.5%)	65 (30.1%)	92 (40.5%)
***Data missing for, (n)***	*37*	*1*	*0*	*1*	*0*	*2*	*9*	*24*
**Alcohol consumption, *n* (%)**								
**≤2 standard drinks/day**	374 (93.3%)	26 (100%)	20 (100%)	40 (90.9%)	48 (94.1%)	58 (96.7%)	60 (93.8%)	122 (89.7%)
**>2 standard drinks/day**	27 (6.7%)	0 (0.0%)	0 (0.0%)	4 (9.1%)	3 (5.9%)	2 (3.3%)	4 (6.3%)	14 (10.3%)
***Data missing for, (n)***	*708*	*57*	*71*	*64*	*94*	*146*	*161*	*115*
**Smoking status, *n* (%)**								
**Never smoked**	525 (60.3%)	68 (81.9%)	45 (59.2%)	60 (63.8%)	68 (60.7%)	63 (46.7%)	87 (57.2%)	134 (61.2%)
**Former smoker**	278 (31.9%)	7 (8.4%)	22 (28.9%)	23 (24.5%)	28 (25.0%)	61 (45.2%)	60 (39.5%)	77 (35.2%)
**Current smoker**	68 (7.8%)	8 (9.6%)	9 (11.8%)	11 (11.7%)	16 (14.3%)	11 (8.1%)	5 (3.3%)	8 (3.7%)
***Data missing for, (n)***	*238*	*0*	*15*	*14*	*33*	*71*	*73*	*32*
**Subjectively measured sleep, *n* (%)**								
**Short-sleep duration <7 hours**	387 (35.3%)	15 (18.1%)	25 (27.5%)	33 (30.6%)	61 (42.4%)	76 (37.1%)	85 (38.3%)	92 (37.9%)
**Normal-sleep duration ≥7 hours and <9 hours**	630 (57.5%)	61 (73.5%)	62 (68.1%)	68 (63.0%)	78 (54.2%)	118 (57.6%)	116 (52.3%)	127 (52.3%)
**Long-sleep duration ≥9 hours**	79 (7.2%)	7 (8.4%)	4 (4.4%)	7 (6.5%)	5 (3.5%)	11 (5.4%)	21 (9.5%)	24 (9.9%)
***Data missing for, (n)***	*13*	*0*	*0*	*0*	*1*	*1*	*3*	*8*
**PROMIS fatigue, mean (SD)**	44.3 (9.2)	42.2 (7.6)	45.9 (8.2)	45.2 (8.9)	44.0 (8.4)	42.1 (9.5)	43.3 (8.9)	47.4 (9.7)
**T-score ≥ 55**	132 (13.1%)	5 (6.3%)	11 (12.6%)	16 (15.4%)	12 (8.7%)	21 (11.0%)	17 (8.3%)	50 (24.9%)
**T-score < 55**	873 (86.9%)	74 (93.7%)	76 (87.4%)	88 (84.6%)	126 (91.3%)	170 (89.0%)	188 (91.7%)	151 (75.1%)
***Data missing for, (n)***	*104*	*4*	*4*	*4*	*7*	*15*	*20*	*50*
**PROMIS social isolation, mean (SD)**	40.3 (7.3)	39.5 (7.3)	40.9 (8.0)	42.0 (7.9)	39.2 (6.7)	39.2 (6.8)	39.6 (7.2)	41.7 (7.3)
**T-score ≥ 55**	45 (4.4%)	2 (2.5%)	7 (8.1%)	6 (5.8%)	2 (1.4%)	5 (2.6%)	12 (6.0%)	11 (5.3%)
**T-score < 55**	968 (95.6%)	78 (97.5%)	79 (91.9%)	97 (94.2%)	139 (98.6%)	189 (97.4%)	189 (94.0%)	197 (94.7%)
***Data missing for, (n)***	*96*	*3*	*5*	*5*	*4*	*12*	*24*	*43*

Data are expressed as mean ± SD or as absolute values/percentages. Missing data from participants (*n*) specified in categories lacking data. Abbreviations: FFQ, Food Frequency Questionnaire; IPAQ, International Physical Activity Questionnaire; PROMIS, Patient-Reported Outcomes Measurement Information System.

a Physical activity included walking, cycling or any other moderate/intense exercise assessed with the IPAQ. Alcohol consumption was assessed using the FFQ. The PROMIS short form 8a questionnaires were used to score fatigue and social isolation with higher T-scores representing more fatigue. T-scores are standardized scores with a mean of 50 and a SD of 10 (interpretation: scores <40 are >1 SD below the mean, and the scores >60 are >1 SD above the mean) with reference to the general population in the United States.

### Intrinsic capacity abnormalities in ICOPE Step 1: screening at baseline

Table 4 shows the prevalence of abnormalities in IC domains identified with ICOPE *Step 1*. screening. There were significant differences in the prevalence of abnormalities across age groups in all IC domains. The most prevalent IC abnormalities identified using ICOPE *Step 1*. appeared to be abnormalities in vision (89.3%), audition (30.5%), and cognition (24.7%) in the total baseline population. Vision abnormalities were the most prevalent IC domain abnormality in the youngest age group (53.7%), and more than 95% of participants aged 50 years and older had a vision abnormality. Abnormalities in audition ranged progressively from 3.6% in the youngest age group to 67.9% in the oldest age-group. Abnormalities in cognition remained low (6%-8.8%) until the age of 50 years old then the number of abnormalities increased to 16.6% ranging progressively to 43.8% in the oldest age-group. Abnormalities in psychological well-being varied between 16.9% and 23.8% below the age of 80-years after which the number of abnormalities appeared to increase to 33.1%. Abnormalities in vitality fell in the range of 1.2% to 7.7% below the age of 80-years with 13.7% of subjects ≥80 years old exhibiting abnormalities in this domain. Abnormalities in locomotor capacity were the least frequent abnormality in IC in the total population (5.4%). Locomotor capacity abnormalities were negligible until the age of 60 years and even in the oldest age-group the prevalence was 13.4%.

**Table 4.** Baseline intrinsic capacity abnormalities in ICOPE *Step 1* screening for the whole cohort and by sex (*n* = 1109).

ICOPE Step 1. abnormalities in order of prevalence in total cohort	Total	20-29 years	30-39 years	40-49 years	50-59 years	60-69 years	70-79 years	≥80 years	*P*-value
	(*n* = 1109)	(*n* = 83)	(*n =* 91)	(*n* = 108)	(*n* = 145)	(*n* = 206)	(*n* = 225)	(*n* = 251)	
**Vision, *n* (%)**	988 (89.3%)	44 (53.7%)	51 (56.0%)	93 (86.1%)	139 (95.9%)	202 (98.1%)	216 (96.0%)	243 (97.2%)	<.001
***Data missing for, (n)***	*2*	*1*	*0*	*0*	*0*	*0*	*0*	*1*	
**Males, *n* (%)**	374 (88.8%)	12 (46.2%)	15 (44.1%)	29 (82.9%)	42 (97.7%)	75 (97.4%)	96 (97.0%)	105 (98.1%)	<.001
***Data missing for, (n)***	*1*	*0*	*0*	*0*	*0*	*0*	*0*	*1*	
**Females, *n* (%)**	614 (89.5%)	32 (57.1%)	36 (63.2%)	64 (87.7%)	97 (95.1%)	127 (98.4%)	120 (95.2%)	138 (96.5%)	<.001
***Data missing for, (n)***	*1*	*1*	*0*	*0*	*0*	*0*	*0*	*0*	
**Audition, *n* (%)**	338 (30.5%)	3 (3.6%)	5 (5.5%)	6 (5.6%)	15 (10.3%)	40 (19.4%)	100 (44.4%)	169 (67.9%)	<.001
***Data missing for, (n)***	*2*	*0*	*0*	*0*	*0*	*0*	*0*	*2*	
**Males, *n* (%)**	162 (38.5%)	3 (11.5%)	2 (5.9%)	2 (5.7%)	7 (16.3%)	19 (24.7%)	50 (50.5%)	79 (73.8%)	<.001
***Data missing for, (n)***	*1*	*0*	*0*	*0*	*0*	*0*	*0*	*1*	
**Females, *n* (%)**	176 (25.7%)	0 (0.0%)	3 (5.3%)	4 (5.5%)	8 (7.8%)	21 (16.3%)	50 (39.7%)	90 (63.4%)	<.001
***Data missing for, (n)***	*1*	*0*	*0*	*0*	*0*	*0*	*0*	*1*	
**Cognition, *n* (%)**	273 (24.7%)	5 (6.0%)	8 (8.8%)	6 (5.6%)	24 (16.6%)	47 (22.8%)	74 (32.9%)	109 (43.8%)	<.001
***Data missing for, (n)***	*2*	*0*	*0*	*0*	*0*	*0*	*0*	*2*	
**Males, *n* (%)**	108 (25.7%)	1 (3.8%)	4 (11.8%)	2 (5.7%)	5 (11.6%)	17 (22.1%)	30 (30.3%)	49 (45.8%)	<.001
***Data missing for, (n)***	*1*	*0*	*0*	*0*	*0*	*0*	*0*	*1*	
**Females, *n* (%)**	165 (24.1%)	4 (7.0%)	4 (7.0%)	4 (5.5%)	19 (18.6%)	30 (23.3%)	44 (34.9%)	60 (42.3%)	<.001
***Data missing for, (n)***	*1*	*0*	*0*	*0*	*0*	*0*	*0*	*1*	
**Psychological well-being, *n* (%)**	264 (24.0%)	14 (16.9%)	21 (23.1%)	25 (23.6%)	26 (18.1%)	43 (21.0%)	53 (23.8%)	82 (33.1%)	.0080
***Data missing for, (n)***	*9*	*0*	*0*	*2*	*1*	*1*	*2*	*3*	
**Males, *n* (%)**	85 (20.3%)	3 (11.5%)	6 (17.6%)	10 (28.6%)	5 (11.6%)	9 (11.8%)	20 (20.4%)	32 (30.2%)	.0235
***Data missing for, (n)***	*4*	*0*	*0*	*0*	*0*	*1*	*1*	*2*	
**Females, *n* (%)**	179 (26.2%)	11 (19.3%)	15 (26.3%)	15 (21.1%)	21 (20.8%)	34 (26.4%)	33 (26.4%)	50 (35.2%)	.1391
***Data missing for, (n)***	*5*	*0*	*0*	*2*	*1*	*0*	*1*	*1*	
**Vitality, *n* (%)**	77 (7.0%)	1 (1.2%)	7 (7.7%)	1 (0.9%)	4 (2.8%)	13 (6.3%)	17 (7.6%)	34 (13.7%)	<.001
***Data missing for, (n)***	*3*	*1*	*0*	*0*	*0*	*0*	*0*	*2*	
**Males, *n* (%)**	27 (6.4%)	0 (0.0%)	1 (2.9%)	0 (0.0%)	1 (2.3%)	5 (6.5%)	8 (8.1%)	12 (11.2%)	.1005
***Data missing for, (n)***	*1*	*0*	*0*	*0*	*0*	*0*	*0*	*1*	
**Females, *n* (%)**	50 (7.3%)	1 (1.8%)	6 (10.5%)	1 (1.4%)	3 (2.9%)	8 (6.2%)	9 (7.1%)	22 (15.5%)	.0004
***Data missing for, (n)***	*2*	*1*	*0*	*0*	*0*	*0*	*0*	*1*	
**Locomotor capacity, *n* (%)**	59 (5.4%)	0 (0.0%)	1 (1.1%)	1 (0.9%)	0 (0.0%)	8 (4.0%)	16 (7.2%)	33 (13.4%)	<.001
***Data missing for, (n)***	*13*	*0*	*0*	*0*	*0*	*4*	*4*	*5*	
**Males, *n* (%)**	20 (4.8%)	0 (0.0%)	0 (0.0%)	0 (0.0%)	0 (0.0%)	2 (2.6%)	6 (6.3%)	12 (11.2%)	.0067
***Data missing for, (n)***	*5*	*0*	*0*	*0*	*0*	*1*	*3*	*1*	
**Females, *n* (%)**	39 (5.7%)	0 (0.0%)	1 (1.8%)	1 (1.4%)	0 (0.0%)	6 (4.8%)	10 (8.0%)	21 (15.1%)	<.001
***Data missing for, (n)***	*8*	*0*	*0*	*0*	*0*	*3*	*1*	*4*	

An abnormality is a deviation from normal in a domain specific question in ICOPE *Step 1* screening. Vision was measured by the following questions: Do you have any problems with your eyes? Difficulties in seeing far, reading, eye diseases or are you currently under medical treatment? A «yes» response determined an abnormality. Audition was measured using the whisper test. The evaluator softly whispers a two-syllable word and asks the person to repeat the word. The evaluator then tests the other ear using a different word. Not repeating the correct words determined an abnormality. Cognition was measured by the three-word remember test and the following four orientation questions: Which year is it? What month is it? What day of the month is it? What day of the week is it? from the MMSE. An abnormality was recorded if an individual was unable to remember at least one word or if they provided an incorrect response to an orientation question. Psychological well-being was measured by the following two questions: Over the past 2 weeks, have you been bothered by: 1. Feeling down, depressed or hopeless? 2. Having little interest or pleasure in doing things? A «yes» response for at least one question determined an abnormality. Vitality was measured by the following two questions: Have you unintentionally lost more than 3 kg over the last 3 months? Have you experienced loss of appetite? A «yes» response for at least one question determined an abnormality. Data are expressed as absolute values/percentages. Locomotor capacity was measured by time (in seconds) required to perform five chair rises. An abnormality is when the time needed to complete the test is ≥14 seconds for subjects <80 years old or ≥16 seconds for subjects ≥80 years old or number of chair rises <5. Differences between age groups (*P*-values for the whole population and by sex) were assessed using chi square tests for categorical variables with *P* < .05 considered as statistically significant. Missing data from participants (*n*) specified in categories lacking data.Abbreviations: ICOPE, Integrated Care for Older People; MMSE, Mini Mental State Examination.

There were also significant differences in abnormalities in all IC domains in sex-stratified analysis with the exception of the vitality domain in males and the psychological well-being domain in females. Although post hoc comparisons between sexes were not performed the descriptive data suggests that the prevalence of ICOPE *Step 1*, screening abnormalities in locomotor capacity were negligible in both males and females below the age of 60 years, after which abnormalities ranged progressively from 2.6% to 11.2% in males and from 4.8% to 15.1% in females (between the ages of 60–80+ years). Below the age of 60-years, the prevalence of abnormalities in cognition varied between 3.8%-11.8% in males and 5.5%–18.6% in females then between the age of 60-80+ years the number of abnormalities ranged progressively from 22.1% to 45.8% in males and from 23.3% to 42.3% in females. Sex differences between age groups seemed apparent for the domains of psychological well-being and audition. Men appeared to exhibit overall better psychological well-being with the exception of men aged between 40 and 49 years, and females seemed to possess better hearing across all age groups. The prevalence of abnormalities in the vitality domain of IC appeared broadly similar across age groups in males and females with the exception of 30–39 year old women who appeared to have more abnormalities in *Step 1* screening than males. In terms of vision, males and females seemed to follow a similar trend in the increase in the prevalence of abnormalities from the age of 40 years with males appearing to have better vision than females between 20 and 39 years old.

### ICOPE Step 2: tests for baseline intrinsic capacity abnormalities

Table 5 shows the results of ICOPE Step 2. baseline assessments for the further characterization of IC. There were significant differences in the mean scores and the presence of abnormalities (determined using the cut-offs described in the *Methods*) in all test of IC domains across age groups except for the domain of psychological well-being with dichotomized data. The most prevalent IC abnormalities in the total population according to ICOPE *Step 2* were those in the domains of vision (24.2%), psychological well-being (22.7%), and locomotor capacity (7.3%). The WHO simple eye chart (vision test) demonstrated that the prevalence of abnormalities in visual acuity ranged progressively from 2.4% in the youngest age-group to 40.1% in the oldest age-group. The mean PHQ-9 score (psychological well-being test) of the total population was 3.1 ± 3.5. Depression was prevalent in 22.7% of the total cohort (PHQ-9 score >4) with depression appearing to be most prevalent in the oldest age-group (28.7%) and least prevalent in the youngest age-group (14.6%). The prevalence of depression varied between 17.2% and 25.8% between these upper and lower limits of age distribution. The mean SPPB score (locomotor capacity test) of the cohort was 11.4 ± 1.6 with 92.7% of the total population exhibiting no mobility limitations (SPPB score ≥10). The percentage of subjects with SPPB score <10 was low (0%-0.7%) in individuals aged between 20 and 59 years. From the age of 60 years, there seemed to be a gradual increase in the percentage of individuals with SPPB score <10 with the percentage of people falling below this cut-off ranging progressively from 3.0% in the 60-69 years group to 24.0% in the oldest age group. The mean MMSE score (cognitive domain test) of the cohort was 28.6 ± 1.9. In individuals aged between 20 and 59 years, the percentage of subjects with MMSE score <26 remained low (0%–1.4%). From the age of 60 years, there seemed to be a progressive increase in the percentage of people with MMSE score <26, suggestive of cognitive impairment, with the percentage of people below this cut-off ranging progressively from: 2.9% in the 60-69 years group to 18.4% in the oldest age group. The mean MNA score (vitality test) of the total cohort was 27.4 ± 2.1 indicative of normal nutritional status (score >23.5/30) with 6.3% of the total population possessing an MNA score ≤23.5 indicating a risk of malnutrition. It appeared that the subjects at greatest risk of malnutrition were those in the oldest age group (prevalence: 10.9%).

**Table 5.** ICOPE *Step 2* tests of intrinsic capacity at baseline (*n* = 1109).

ICOPE Step 2. Abnormalities in order of prevalence in total cohort	Total	20-29 years	30-39 years	40-49 years	50-59 years	60-69 years	70-79 years	≥80 years	*P*-value
	(*n* = 1109)	(*n* = 83)	(*n* = 91)	(*n* = 108)	(*n* = 145)	(*n* = 206)	(*n* = 225)	(*n* = 251)	
**Vision**									
**WHO simple eye chart (anomaly), *n* (%)**	264 (24.2%)	2 (2.4%)	4 (4.4%)	8 (7.5%)	31 (21.4%)	46 (22.7%)	76 (34.4%)	97 (40.1%)	<.001^b^
***Data missing for, (n)***	*17*	*0*	*0*	*1*	*0*	*3*	*4*	*9*	
**Depression (psychological well-being)**									
**PHQ-9 (score/27), mean (SD)**	3.1 (3.5)	2.4 (2.7)	3.1 (2.6)	3.1 (3.6)	2.6 (3.1)	2.8 (3.4)	3.1 (3.4)	3.9 (4.4)	.0109^a^
**PHQ-9 > 4 (abnormality)**	249 (22.7%)	12 (14.6%)	23 (25.8%)	25 (23.1%)	25 (17.2%)	44 (21.7%)	49 (22.0%)	71 (28.7%)	.0782^b^
**PHQ-9 ≤ 4**	848 (77.3%)	70 (85.4%)	66 (74.2%)	83 (76.9%)	120 (82.8%)	159 (78.3%)	174 (78.0%)	176 (71.3%)	
***Data missing for, (n)***	*12*	*1*	*2*	*0*	*0*	*3*	*2*	*4*	
**Physical performance (locomotor capacity)**									
**SPPB (score/12), mean (SD)**	11.4 (1.6)	12.0 (0.2)	12.0 (0.2)	11.9 (0.3)	11.9 (0.4)	11.8 (0.7)	11.4 (1.5)	10.2 (2.6)	<.001^a^
**SPBB < 10 (abnormality)**	79 (7.3%)	0 (0.0%)	0 (0.0%)	0 (0.0%)	1 (0.7%)	6 (3.0%)	14 (6.5%)	58 (24.0%)	<.001^b^
**SPBB ≥ 10**	1 005 (92.7%)	83 (100%)	91 (100%)	108 (100%)	143 (99.3%)	194 (97.0%)	202 (93.5%)	184 (76.0%)	
***Data missing for, (n)***	*25*	*0*	*0*	*0*	*1*	*6*	*9*	*9*	
**Cognition**									
**MMSE (score/30), mean (SD)**	28.6 (1.9)	29.4 (1.0)	29.2 (1.0)	29.3 (0.9)	29.0 (1.2)	28.8 (1.3)	28.5 (1.7)	27.4 (3.0)	<.001^a^
**MMSE < 26 (abnormality)**	69 (6.3%)	1 (1.2%)	1 (1.1%)	0 (0.0%)	2 (1.4%)	6 (2.9%)	14 (6.3%)	45 (18.4%)	<.001^b^
**MMSE ≥ 26**	1 032 (93.7%)	82 (98.8%)	90 (98.9%)	108 (100%)	143 (98.6%)	200 (97.1%)	209 (93.7%)	200 (81.6%)	
***Data missing for, (n)***	*8*	*0*	*0*	*0*	*0*	*0*	*2*	*6*	
**Nutritional status (vitality)**									
**MNA (score/30), mean (SD)**	27.4 (2.1)	26.9 (2.1)	26.9 (2.1)	27.2 (1.6)	27.6 (1.8)	27.8 (1.7)	27.7 (2.1)	27.0 (2.7)	<.001^a^
**MNA ≤ 23.5 (abnormality)**	70 (6.3%)	6 (7.2%)	6 (6.6%)	4 (3.7%)	7 (4.8%)	9 (4.4%)	11 (4.9%)	27 (10.9%)	.0458^b^
**MNA > 23.5**	1034 (93.7%)	77 (92.8%)	85 (93.4%)	104 (96.3%)	138 (95.2%)	197 (95.6%)	213 (95.1%)	220 (89.1%)	
***Data missing for, (n)***	*5*	*0*	*0*	*0*	*0*	*0*	*1*	*4*	

Vision abnormalities were assessed using the WHO simple eye chart for the assessment of general visual acuity. Depression/psychological well-being was assessed using the PHQ-9 questionnaire with higher scores associated with a higher risk of depression. A PHQ-9 > 4 was considered as an abnormality. Physical performance was assessed using the SPPB with higher scores indicating better mobility. An SPBB < 10 was considered as an abnormality in locomotor capacity. Cognition was assessed using the MMSE with higher scores indicating better cognition. An MMSE < 26 was considered as an abnormality. Nutritional status was assessed using the MNA with higher scores reflecting better nutritional status. An MNA score of ≤23.5 was considered as an abnormality. Data are expressed as mean ± SD or as absolute values/percentages. Missing data from participants (*n*) specified in categories lacking data.Abbreviations: ICOPE, Integrated Care for Older People; MMSE, Mini Mental State Examination; MNA, Mini Nutritional Assessment; PHQ-9, Patient Health Questionnaire; SPPB, Short Physical Performance Battery.

a Differences between age groups for the whole population were assessed using Kruskal-Wallis for continuous variables with *p* < 0.05 considered as statistically significant.

b Differences between age groups for the whole population were assessed using chi square tests for categorical variables with *p* < 0.05 considered as statistically significant.

## Conclusions

To the best of our knowledge, INSPIRE-T represents the largest and longest research cohort study for the investigation of IC. Data on IC was collected at study baseline in accordance with the WHO ICOPE program (*Steps 1 and 2*). We observed that abnormalities in many domains of IC were higher in prevalence from around the age of 60 years, consistent with the intended use of ICOPE in older adults.^31^ The most prevalent IC abnormalities identified using ICOPE *Step 1* appeared to be in the sensory and cognitive domains in the total cohort. Indeed, sensory deficits can lead to social isolation, which is a risk factor for cognitive decline.^32^ Moreover, hearing loss is directly associated with cognitive decline and Alzheimer’s disease (AD).^33^ Thus, these deficits might be interconnected. Abnormalities in locomotor capacity appeared to be the least frequent abnormality even in the oldest age group the prevalence was low, suggestive of a lower risk of falls and likelihood of dependency.^3^

More research is required before definite conclusions can be drawn, but trends seemed comparable between males and females in the prevalence of ICOPE *Step 1* abnormalities in locomotor capacity and cognition from the age of 60 years. Previous studies on sex differences in physical performance/locomotor capacity with age highlight mixed results with no sex-differences across adulthood^34^ and worse performance in older women^35^^,^^36^ being reported. Furthermore, evidence suggests that sex does not determine the rate of cognitive decline at least between 60 and 80 years of age.^37^ Nevertheless, AD is more prevalent in women potentially attributable to hormonal influences,^38^ longer life-span and survivorship bias among men.^39^ Sex differences between age groups were observed in psychological well-being and audition with men seeming to possess better psychological well-being independent of age with the exception of men aged between 40 and 49 years and females appeared to have better audition *per se*. Accordingly, research has indicated that depression is more common in women than men,^40^ possibly due to hormonal fluctuations in women.^41^ Moreover, a peak in the prevalence of depression has been identified during middle adulthood (45-59 years old) particularly in men possibly attributable to increased economic, familial or social pressures/roles.^42^ It has also been previously reported that women have more sensitive hearing than men^43^ and men experience earlier age-related hearing loss (presbycusis) than females.^44^^,^^45^ The causes of presbycusis are multifaceted, but hormonal differences might account, at least in part, for sex differences in its onset.^45^ Similar trends in the prevalence of abnormalities in vitality were apparent between sexes across age groups apart from 30-39 year old women who appeared to have a greater prevalence of abnormalities compared to their male counterparts. This might reflect nutritional deficits linked to the demands of childbearing. Indeed, energy intake, macronutrients,^46^ and some vitamins and minerals such as vitamin D, folate, and iron are often suboptimal in pregnant women.^47^ From the age of 40 years old, both sexes seemed to follow similar ascending trajectories in the accumulation of vision abnormalities, consistent with the onset of presbyopia,^48^ while, males appeared to have better vision than females between the ages of 20 and 39 years. Accordingly, previous findings also suggest that women aged between 20 and 45 years old exhibit a greater prevalence of refractive errors (myopia and hyperopia) than men.^49^ Furthermore, women are more likely to report health symptoms and seek medical help than males^50^ and are thus more likely to respond with a “yes” response to the ICOPE *Step 1* vision question.

The ICOPE *Step 2* tests (according to the cut-offs described to determine an abnormality) suggested that the most pre­valent IC abnormalities in the total population were those in the domains of vision (24.2% vs. 89.3% with ICOPE *Step 1*), psychological well-being (22.7% vs. 24% with ICOPE *Step 1*), and locomotor capacity (7.3% vs. 5.4% with ICOPE *Step 1*). However, it should be noted that differences between age groups in psychological well-being abnormalities were not statistically different according to ICOPE *Step 2* testing when PHQ-9 scores were dichotomized with a positivity threshold of >4, whereas in ICOPE *Step 1* testing and when comparing mean PHQ-9 scores significant differences between age groups were observed. ICOPE *Step 2* testing suggested that the least prevalent IC abnormalities in the total population were those in the domains of cognition (6.3% vs. 24.7% with ICOPE *Step 1*) and vitality (6.3% vs. 7.0% with ICOPE *Step 1*).

The ranges for abnormalities in psychological well-being screening in ICOPE *Step 1* (16.9%-33.1%) and ICOPE *Step 2* (14.6%–28.7%) tests were broadly similar. Likewise, the ranges for abnormalities in vitality screening in ICOPE *Step 1* (0.9%-13.7%) and ICOPE *Step 2* (3.7%-10.9%) were comparable. However, the ranges of abnormalities in vision screening in ICOPE *Step 1* (53.7%-98.1%) and ICOPE *Step 2* (2.4%-40.1%) tests appeared very different. Similar discrepancies in the ranges of abnormalities were seen with cognition screening in ICOPE *Step 1* (5.6%-43.8%) and ICOPE *Step 2* (0%-18.4%) tests. The ranges in abnormalities for locomotor capacity identified with ICOPE *Step 1* (0%-13.4%) and ICOPE *Step 2* (0%-24%) screening were also discrepant with the disparity seemingly apparent only in the oldest age group. Further in-depth research is required to explore whether ICOPE *Step 1* and *Step 2* screening sensitivity could be optimized by adapting tests and adjusting abnormality positivity thresholds, while maintaining feasibility as brief clinical tools. Aligned with the goal of developing more sensitive measures, supplementary tests (including the MAPT-PACC score for detecting early cognitive loss) to explore IC and its underlying biology were performed in INSPIRE-T (see Supplementary material). Future investigation will determine whether some of these tests might be more sensitive to detect domain-specific changes in IC and thus represent potential candidate tests for incorporation into the ICOPE screening pathway.

The INSPIRE-T study has generated a broad-ranging biological sample set consisting of blood, urine, saliva, dental plaque, nasopharyngeal swabs, stools, hair follicles, skin surface samples, and skin biopsies. The biobank represents a unique, shared-resource platform that will help drive scientific research complimenting other large biorepositories such as the UK Biobank^51^ and the China Kadoorie Biobank.^52^ A longer-term aim of INSPIRE-T is to identify novel biomarkers of IC declines, therefore biospecimens will be subjected to exploratory biomarker analysis using multiomics as well as target-specific biomarker investigation (*incl*. ATPase inhibitory factor 1 and apelin amongst others). Leveraging these results, we plan to devise an IC clock consisting of a panel of biomarkers in order to simultaneously capture the diverse physiological signatures of aging. This will facilitate the sensitive measurement and monitoring of biological versus chronological IC status and complement our recently developed epigenetic IC clock.^53^ Furthermore, we aim to identify potential drug candidates for the prevention of IC declines that will inform the design of human clinical trials. Thus, the ultimate goal of INSPIRE-T is to enable predictive, preventive, and personalized medicine for the maintenance of IC.

Other large longitudinal observational studies are being conducted for the examination of general health conditions such as “CONSTANCES”^54^ and “All of Us.”^55^ Large well-established studies of aging also exist and include the Baltimore Longitudinal Study of Aging^56^ as well as disease-specific cohort studies such as the Framingham Heart Study^57^ and the Alzheimer's Disease Neuroimaging initiative (ADNI).^58^ INSPIRE-T adds to these existing studies and is unique in design through its specific capture of IC longitudinally. INSPIRE-T moves away from the specific study of age-related diseases and centers on the assessment and maintenance of global function with age. This facet of INSPIRE-T is particularly relevant considering that life expectancy has risen significantly, while healthspan has not correspondingly increased.^59^ This disparity between lifespan and healthspan has been attributed to the many chronic diseases afflicting older adults resulting in multisystem decline and poor health.^59^ Thus, the increase in longevity demands new health initiatives in order to identify and subvert declines in global physiological/psychological function thereby promoting healthy aging. The new data generated by INSPIRE-T focused on IC will help narrow this lifespan—healthspan gap and in turn reduce economic healthcare burden.

The strengths of INSPIRE-T include the follow-up of 10 years, the diverse biospecimen collection and the plethora of clinical assessments performed. The assessments were also conducted in one research-center circumventing inter-site variability in data collection. A key strength of INSPIRE-T is its emphasize on data and biospecimen sharing, which will promote collaboration and expediate scientific discovery. The limitations of INSPIRE-T include the fact that the study sample does not represent the general population in terms of the high educational level exhibited by the subjects and the lack of participants with less favorable socioeconomic status. New recruitment strategies are, however, currently in operation to address the bias in the study population in terms of demographic variables through oversampling of under-represented groups. Another limitation of the study is that there appears to be ceiling effects in the data capture particularly for ICOPE *Step 1* screening for locomotor capacity and the assessment of cognition using the MMSE in ICOPE *Step 2* as well as floor effects in ICOPE *Step 1* vision screening. This could reflect under/over sensitivity in the assessments and/or the high physical and cognitive health status of the cohort.

In summary, we present here the baseline data for the INSPIRE-T longitudinal observational study. This data provides a significant step toward a better understanding of the aging process centered on IC and the maintenance of global function. This is in line with the WHO’s first world report on ageing and health that describes IC as the new framework for defining healthy aging.^1^ Future studies using the next waves of the INSPIRE-T data collection will be pivotal for capturing IC trajectories and exploring the biological mechanisms underlying aging particularly as a function of lifestyle characteristics and health conditions.

***The members of the IHU HealthAge INSPIRE/Open Science Group are:***

***IHU HealthAge INSPIRE Platform Group:***

*Inspire-T human cohort group: Coordinators:* Sophie Guyonnet, Bruno Vellas; *Project managers:* Lauréane Brigitte, Agathe Milhet; *Clinical Research Assistants*: Elodie Paez, Emeline Muller, Sabine Le Floch; *Investigators:* Catherine Takeda, Catherine Faisant, Françoise Lala, Gabor Abellan Van Kan, Zara Steinmeyer, Antoine Piau, Tony Macaron, Davide Angioni, Pierre-Jean Ousset; *Nurses:* Mélanie Comté, Nathalie Daniaud, Fanny Boissou-Parachaud; *Methodology, statistical analysis and data management subgroup:* Sandrine Andrieu, Christelle Cantet; *Body composition, VO_2_max, isocinetism subgroup*: Yves Rolland, Philipe de Souto Barreto, Fabien Pillard; Technician DXA—Bernard Teysseyre; *MRI subgroup:* Marie Faruch, Pierre Payoux; *ICOPE subgroup:* Catherine Takeda, Neda Tavassoli; *Biological sample collection subgroup:* Marie Dorard, Bénédicte Razat, Camille Champigny, Sophie Guyonnet

*Inspire animal cohort groups:* Cédric Dray, Jean-Philippe Pradère (Fish colony); Angelo Parini, Yohan Santin (Murine cohort)

*Associated research teams:* Dominique Langin, Pierre Gourdy, Laurent O. Martinez, Anne Bouloumié, Angelo Parini (I2MC lab); Nicolas Fazilleau, Roland Liblau, Jean-Charles Guéry, Michel Simon, Nicolas Gaudenzio, Luciana Bostan, Hicham El Costa, Nabila Jabrane Ferrat (Infinity lab); Philippe Valet, Cedric Dray, Isabelle Ader, Valérie Planat, Louis Casteilla (Restore); Pierre Payoux, Patrice Peran (Tonic lab); Cyrille Delpierre, Sandrine Andrieu (CERPOP lab); Claire Rampon, Noelie Davezac, Bruno Guiard (CRCA/CBI lab); Nathalie Vergnolle, Jean-Paul Motta, Sara Djebali, Pauline Floch, Céline Deraison, Chrystelle Bonnart (IRSD lab); Jean-Emmanuel Sarry (CRCT lab);

***IHU HealthAge Open Science group***: Nicola Coley, Sophie Guyonnet, Sandrine Andrieu (contact: ihuos_inspiredataaccess@chu-toulouse.fr).

## Supplementary Material

glaf181_Supplementary_Data

## Data Availability

De-identified data from the INSPIRE-T cohort are available to researchers upon request, following approval of a methodologically sound research proposal by the INSPIRE-T data access committee and signature of a data use agreement. Enquiries or proposals should be addressed to ihuos_inspiredataaccess@chu-toulouse.fr.
