# Supplementary material for: The INSPIRE-T longitudinal observational study centered on intrinsic capacity: baseline data
Source: J Gerontol A Biol Sci Med Sci. 2025 Aug 19;80(9):glaf181. doi: 10.1093/gerona/glaf181 (PMC12448789; doi:10.1093/gerona/glaf181)
Supplement: glaf181_Supplementary_Data [file glaf181_supplementary_data.pdf]

The INSPIRE-T longitudinal observational study centred on intrinsic capacity: baseline data

Guyonnet et al., 2025

Supplementary material contents:

eTable 1. Other baseline tests to explore intrinsic capacity and its underlying biology (n = 1109)

etable 2. Baseline frailty phenotype presented for the total population and by sex (n = 1109)

eTable 3. Baseline Dual-energy X-ray absorptiometry measurements for body composition and the assessment of sarcopenia presented for the total population and by sex (n = 850)

eTable 4. Baseline maximal oxygen consumption (VO_2 max) and isokinetic muscle strength (Cybex) (n = 279 and 286 respectively)

eTable 5. Cognitive data for participants < 70 years old at baseline (n = 633)

eTable 6. Biospecimens collected at baseline constituting the INSPIRE-T biobank

eTable 1. Other baseline tests to explore intrinsic capacity and its underlying biology (n = 1109)

	Total (n = 1109)	20-29 years (n = 83)	30-39 years (n = 91)	40-49 years (n = 108)	50-59 years (n = 145)	60-69 years (n = 206)	70-79 years (n = 225)	≥ 80 years (n = 251)
**CFI (score/14), mean (SD)**	2.3 (2.4)	0.9 (1.2)	1.3 (1.6)	1.8 (2.4)	1.8 (2.0)	2.0 (1.9)	2.9 (2.5)	4.1 (2.8)
Data missing for, (n)	*212*	*6*	*5*	*8*	*5*	*26*	*52*	*110*
**Functional performance**								
ADL (score/6), mean (SD)	5.9 (0.3)	6.0 (0.1)	6.0 (0.1)	6.0 (0.1)	6.0 (0.1)	5.9 (0.2)	5.8 (0.4)	5.7 (0.5)
ADL score = 6	925 (83.6 %)	82 (98.8 %)	88 (96.7 %)	104 (96.3 %)	137 (94.5 %)	182 (88.3 %)	170 (75.6 %)	162 (65.1 %)
ADL < 6	182 (16.4 %)	1 (1.2 %)	3 (3.3 %)	4 (3.7 %)	8 (5.5 %)	24 (11.7 %)	55 (24.4 %)	87 (34.9 %)
Data missing for, (n)	*2*	*0*	*0*	*0*	*0*	*0*	*0*	*2*
IADL (score/8), n (%) ≥ 1 limitation	132 (12.7 %)	1 (1.2 %)	0 (0.0 %)	4 (3.7 %)	3 (2.2 %)	11 (5.6 %)	25 (12.4 %)	88 (38.6 %)
Data missing for, (n)	*68*	*0*	*1*	*1*	*8*	*11*	24	*23*
**PROMIS mobility (T-score), mean (SD)**	53.5 (8.1)	58.2 (4.1)	58.2 (4.2)	56.6 (6.1)	56.6 (5.7)	55.1 (6.3)	51.5 (8.5)	45.5 (8.5)
Data missing for, (n)	*116*	*2*	*4*	*3*	*3*	*13*	*24*	*67*
**Chair rises in 30 seconds, mean (SD)**	15.8 (4.7)	18.8 (4.5)	19.1 (5.5)	18.3 (5.1)	16.5 (3.9)	15.5 (3.6)	14.7 (3.8)	12.6 (3.5)
Data missing for, (n)	*288*	*0*	*16*	*14*	*36*	*77*	*84*	*61*
**Amsler grid (anomaly), n (%)**	61 (5.6 %)	1 (1.2 %)	3 (3.3 %)	0 (0.0 %)	1 (0.7 %)	12 (5.9 %)	15 (6.8 %)	29 (12.0 %)
Data missing for, (n)	*16*	*0*	*0*	*1*	*1*	*2*	*3*	*9*
**Oral status**								
OHAT (score/16), mean (SD)	0.9 (1.4)	0.3 (0.6)	0.6 (1.1)	0.5 (0.9)	0.5 (1.0)	1.0 (1.5)	1.4 (1.6)	1.3 (1.6)
OHAT score 0-3	1033 (94.3 %)	81 (100 %)	87 (95.6 %)	106 (99.1 %)	138 (97.2 %)	197 (96.6 %)	199 (88.8 %)	225 (91.1 %)
OHAT score 4-7	59 (5.4 %)	0 (0.0 %)	4 (4.4 %)	1 (0.9 %)	4 (2.8 %)	6 (2.9 %)	23 (10.3 %)	21 (8.5 %)
OHAT score ≥ 8	4 (0.4 %)	0 (0.0 %)	0 (0.0 %)	0 (0.0 %)	0 (0.0 %)	1 (0.5 %)	2 (0.9 %)	1 (0.4 %)
Data missing for, (n)	*13*	*2*	*0*	*1*	*3*	*2*	*1*	*4*

Notes: The CFI was used for the subjective assessment of cognition with higher scores indicative of worse cognition. Functional performance was assessed using ADL and IADL scales with higher scores indicating greater functional independence. A custom 14 items questionnaire from the PROMIS mobility v2.1 items bank was used to rate locomotor capacity with lower T-scores representing less mobility. T-scores are standardized scores with a mean of 50 and a standard deviation (SD) of 10 (interpretation: scores < 40 are more than 1 SD below the mean and scores > 60 are more than 1 SD above the mean) with reference to the general population in the United States. The chair rise test (number of rises in 30 seconds) was also used to assess locomotor capacity with higher scores indicating better mobility/locomotion. The Amsler grid was used for the assessment of macular health with binary outcomes (normal/anomaly). Oral health status was assessed using the OHAT with higher scores indicative of worse oral health. Data is expressed as mean ± standard deviation (SD) or as absolute values/percentages. Missing data from participants (n) specified in categories lacking data. ***Abbreviations***: ADL, Activities of Daily Living; CFI, the Cognitive Function Instrument; IADL, Instrumental Activities of Daily Living; OHAT, Oral Health Assessment Tool; PROMIS, Patient-Reported Outcomes Measurement Information System; SD, standard deviation.

eTable 2. Baseline frailty phenotype presented for the total population and by sex (n = 1109)

**Frailty status total population**	**Total (n = 1109)**	**20-29 years (n = 83)**	**30-39 years (n = 91)**	**40-49 years (n = 108)**	**50-59 years (n = 145)**	**60-69 years (n = 206)**	**70-79 years (n = 225)**	**≥ 80 years (n = 251)**
Robust, n (%)	694 (62.9 %)	65 (78.3 %)	70 (76.9 %)	75 (69.4 %)	108 (74.5 %)	148 (71.8 %)	149 (66.2 %)	79 (32.2 %)
Prefrail, n (%)	348 (31.6 %)	18 (21.7 %)	21 (23.1 %)	31 (28.7 %)	37 (25.5 %)	55 (26.7 %)	66 (29.3 %)	120 (49.0 %)
Frail, n (%)	61 (5.5 %)	0 (0.0 %)	0 (0.0 %)	2 (1.9 %)	0 (0.0 %)	3 (1.5 %)	10 (4.4 %)	46 (18.8 %)
Data missing for, (n)	*6*	*0*	*0*	*0*	*0*	*0*	*0*	*6*
**Frailty status female**	**Total (n = 687)**	**20-29 years (n = 57)**	**30-39 years (n = 57)**	**40-49 years (n = 73)**	**50-59 years (n = 102)**	**60-69 years (n = 129)**	**70-79 years (n = 126)**	**≥ 80 years (n = 143)**
Robust, n (%)	422 (61.9 %)	45 (78.9 %)	40 (70.2 %)	47 (64.4 %)	71 (69.6 %)	92 (71.3 %)	83 (65.9 %)	44 (31.9 %)
Prefrail, n (%)	217 (31.8 %)	12 (21.2 %)	17 (29.8 %)	24 (32.9 %)	31 (30.4 %)	35 (27.1 %)	37 (29.4 %)	61 (44.2 %)
Frail, n (%)	43 (6.3 %)	0 (0.0 %)	0 (0.0 %)	2 (2.7 %)	0 (0.0 %)	2 (1.6 %)	6 (4.8 %)	33 (23.9 %)
Data missing for, (n)	*5*	*0*	*0*	*0*	*0*	*0*	*0*	*5*
**Frailty status male**	**Total (n = 422)**	**20-29 years (n = 26)**	**30-39 years (n = 34)**	**40-49 years (n = 35)**	**50-59 years (n = 43)**	**60-69 years (n = 77)**	**70-79 years (n = 99)**	**80-89 years (n = 108)**
Robust, n (%)	272 (64.6 %)	20 (76.9 %)	30 (88.2 %)	28 (80.0 %)	37 (86.0 %)	56 (72.7 %)	66 (66.7 %)	35 (32.7 %)
Prefrail, n (%)	131 (31.1 %)	6 (23.1 %)	4 (11.8 %)	7 (20.0 %)	6 (14.0 %)	20 (26.0 %)	29 (29.3 %)	59 (55.1 %)
Frail, n (%)	18 (4.3 %)	0 (0.0 %)	0 (0.0 %)	0 (0.0 %)	0 (0.0 %)	1 (1.3 %)	4 (4.0 %)	13 (12.1 %)
Data missing for, (n)	*1*	*0*	*0*	*0*	*0*	*0*	*0*	*1*

Notes: Frailty was assessed according to Fried's criteria resulting in the designation of a frailty status as either robust, pre-frail or frail according to the number of Fried criteria present. Robust, 0 criteria; pre-frail, 1-2 criteria and frail ≥ 3 criteria. The criteria included: unintentional weight loss, poor handgrip strength, self-reported exhaustion, slow walking speed and low self-reported physical activity.

eTable 3. Baseline Dual-energy X-ray absorptiometry measurements for body composition and the assessment of sarcopenia presented for the total population and by sex (n = 850)

**DXA total population**	**Total (n = 180)**	**20-29 years (n = 73)**	**30-39 years (n = 80)**	**40-49 years (n = 86)**	**50-59 years (n = 124)**	**60-69 years (n = 159)**	**70-79 years (n = 171)**	**≥ 80 years (n = 157)**
**Fat mass (kg), mean (SD)**	22.4 (8.4)	18.8 (7.5)	21.5 (10.7)	20.1 (7.9)	23.4 (8.5)	22.4 (8.0)	24.1 (8.4)	23.3 (7.2)
**Lean mass (kg), mean (SD)**	44.0 (9.3)	43.9 (9.2)	46.8 (10.3)	44.2 (8.4)	45.0 (9.4)	44.0 (9.3)	43.7 (9.5)	42.1 (8.7)
No missing data								
**Sarcopenia, n (%)**	43 (5.1 %)	2 (2.7 %)	6 (7.5 %)	3 (3.5 %)	1 (0.8 %)	4 (2.5 %)	8 (4.7 %)	19 (12.1 %)
Data missing for, (n)	*1*	*0*	*0*	*0*	*0*	*1*	*0*	*0*
**DXA female**	**Total (n = 533)**	**20-29 years (n = 49)**	**30-39 years (n = 51)**	**40-49 years (n = 62)**	**50-59 years (n = 89)**	**60-69 years (n = 100)**	**70-79 years (n = 99)**	**≥ 80 years (n = 83)**
**Fat mass (kg), mean (SD)**	22.8 (8.5)	19.8 (7.4)	21.6 (9.9)	20.3 (7.7)	23.8 (8.4)	23.3 (8.5)	24.5 (8.9)	23.4 (7.5)
**Lean mass (kg), mean (SD)**	38.1 (4.6)	38.4 (4.0)	40.6 (4.6)	39.9 (4.3)	39.7 (3.8)	37.8 (4.1)	36.8 (4.7)	35.2 (4.1)
No missing data								
**Sarcopenia, n (%)**	24 (4.5 %)	0 (0.0 %)	4 (7.8 %)	2 (3.2 %)	1 (1.1 %)	3 (3.0 %)	3 (3.0 %)	11 (13.3 %)
Data missing for, (n)	*1*	*0*	*0*	*0*	*0*	*1*	*0*	*0*
**DXA male**	**Total (n = 317)**	**20-29 years (n = 24)**	**30-39 years (n = 29)**	**40-49 years (n = 24)**	**50-59 years (n = 35)**	**60-69 years (n = 59)**	**70-79 years (n = 72)**	**80-89 years (n = 74)**
**Fat mass (kg), mean (SD)**	21.8 (8.2)	16.6 (7.5)	21.4 (12.2)	19.4 (8.5)	22.7 (8.8)	20.8 (7.0)	23.6 (7.6)	23.2 (7.0)
**Lean mass (kg), mean (SD)**	53.9 (6.5)	55.0 (6.3)	57.8 (8.2)	55.2 (6.1)	58.4 (4.8)	54.4 (5.6)	53.1 (5.7)	49.8 (5.5)
**Sarcopenia (*), n (%)**	19 (6.0 %)	2 (8.3 %)	2 (6.9 %)	1 (4.2 %)	0 (0.0 %)	1 (1.7 %)	5 (6.9 %)	8 (10.8 %)
No missing data								

Notes: Data is expressed as mean ± standard deviation (SD) or as absolute values/percentages. Missing data from participants (n) specified in categories lacking data. (*) Sarcopenia was measured as appendicular lean mass/height^2 (kg/m^2) and defined for women as < 5.5 kg/m^2 and for men as < 7kg/ m^2. ***Abbreviations***: DXA, Dual-energy X-ray absorptiometry. SD, standard deviation.

eTable 4. Baseline maximal oxygen consumption (VO_2 max) and isokinetic muscle strength (Cybex) (n = 279 and 286 respectively)

**Clinical test**	**Total (n = 279)**	**20-29 years (n = 12)**	**30-39 years (n = 21)**	**40-49 years (n = 24)**	**50-59 years (n = 37)**	**60-69 years (n = 80)**	**70-79 years (n = 74)**	**≥ 80 years (n = 31)**
**VO_2 max (ml/kg/min), mean (SD)**	25.6 (8.4)	40.3 (9.4)	31.0 (8.6)	30.9 (8.3)	27.9 (8.2)	25.3 (6.8)	21.5 (6.0)	20.0 (3.9)
Data missing for, (n)	*2*	*0*	*0*	*0*	*0*	*1*	*1*	*0*
	**Total (n = 286)**	**20-29 years (n = 12)**	**30-39 years (n = 22)**	**40-49 years (n = 26)**	**50-59 years (n = 37)**	**60-69 years (n = 81)**	**70-79 years (n = 75)**	**80-89 years (n = 33)**
**Isokinetic muscle strength (Nm), mean (SD)**	94.9 (40.3)	109.9 (33.6)	137.1 (53.9)	109.2 (40.3)	96.9 (36.1)	94.4 (37.1)	88.8 (34.3)	62.2 (23.6)
Data missing for, (n)	*3*	*0*	*0*	*0*	*0*	*1*	*2*	*0*

Notes: Maximal oxygen consumption (VO_2 max) and isokinetic muscle strength test (Cybex) were performed in a subset of participants (n = 279 and n = 286 respectively) between baseline and 24 months. Data is expressed as mean ± standard deviation (SD). Missing data from participants (n) specified in categories lacking data.

eTable 5. Cognitive data for participants < 70 years old at baseline (n = 633)

Cognitive test	Total (n = 633)	20-29 years (n = 83)	30-39 years (n = 91)	40-49 years (n = 108)	50-59 years (n = 145)	60-69 years (n = 206)
**Free and total recall FCSRT (score/96), mean (SD)**	80.6 (8.0)	85.4 (6.5)	83.6 (7.4)	82.4 (6.9)	79.9 (7.1)	76.7 (8.2)
Data missing for, (n)	*9*	*1*	*1*	*3*	*0*	*4*
**Orientation MMSE (score/10), mean (SD)**	10.0 (0.2)	10.0 (0.2)	9.9 (0.3)	10.0 (0.2)	10.0 (0.2)	10.0 (0.2)
Data missing for, (n)	*0*	*0*	*0*	*0*	*0*	*0*
**DSST (symbols/90 seconds), mean (SD)**	59.7 (12.3)	69.7 (9.3)	67.7(10.6)	63.2(11.5)	58.1(9.7)	51.5(10.3)
Data missing for, (n)	*9*	*1*	*1*	*3*	*0*	*4*
**CNT (number of words/2 minutes), mean (SD)**	36.1 (8.8)	38.9 (8.3)	36.4 (7.2)	38.5 (10.5)	35.5 (8.2)	34.0 (8.6)
Data missing for, (n)	*11*	*1*	*1*	*4*	*1*	*4*
**MAPT-PACC, mean (SD)**	-0.0 (0.6)	0.4 (0.5)	0.2 (0.6)	0.2 (0.6)	-0.1 (0.5)	-0.3 (0.6)
Data missing for, (n)	*13*	*1*	*1*	*4*	*1*	*6*

Notes: For participants < 70 years old to assess cognition, the free and total recall of the Free and Cued Selective Reminding Test (FCSRT), the ten MMSE orientation items, the Digit Symbol Substitution Test (DSST) from the Wechsler Adult Intelligence Scale-Revised and the Category Naming Test (CNT) (2-minute category fluency in animals) was performed. Cognition is also represented in this subset as a composite cognitive score known as the MAPT Preclinical Alzheimer Cognitive Composite (MAPT-PACC), which is the mean of the z-scores of the free and total recall of the FCSRT, the ten MMSE orientation items, the DSST and the CNT (an initial estimate of the minimal clinically important difference of this score is - 0·3 points in a year). Data is expressed as mean ± standard deviation (SD). Missing data from participants (n) specified in categories lacking data.
Abbreviations: CNT, Category Naming Test; DSST, Digit Symbol Substitution Test; FCSRT, Free and Cued Selective Reminding test; Orientation MMSE, orientation Mini Mental State Examination; MAPT-PACC, MAPT Preclinical Alzheimer Cognitive Composite; SD, standard deviation.

eTable 6. Biospecimens collected at baseline constituting the INSPIRE-T biobank

Type of sample	Number of subjects
**Dental plaque**	
Upper anterior teeth	691
Upper posterior teeth	695
Lower anterior teeth	694
Lower posterior teeth	694
**Saliva**	757
**Urine**	1106
**Blood**	
Plasma heparin/lithium	930
Plasma EDTA	979
PAXgene Blood DNA tube	640
Red blood cells	936
Whole blood	1117
Serum	1118
**Peripheral blood mononuclear cells (PBMCs)**	1102
**Skin biopsies**	33
**Fibroblasts**	133
**Skin surface samples**	
D’SQUAME® exposed area (corneocytes)	582
D’SQUAME® non-exposed area (corneocytes)	580
Skin swab exposed area	563
Skin swab non-exposed area	561
**Hair follicles**	769
**Stools**	512
**Nasopharyngeal swabs**	202

Notes: Blood, urine, saliva, dental plaque, nasopharyngeal swabs, stools, hair follicles, skin surface samples and skin biopsies were collected from participants (n) at baseline.
